# Interactions between predation and disturbances shape prey communities

**DOI:** 10.1038/s41598-018-21219-x

**Published:** 2018-02-14

**Authors:** Canan Karakoç, Viktoriia Radchuk, Hauke Harms, Antonis Chatzinotas

**Affiliations:** 1 Department of Environmental Microbiology, Helmholtz Centre for Environmental Research - UFZ, Permoserstrasse 15, 04318 Leipzig, Germany; 2 Department of Ecological Dynamics, Leibniz Institute for Zoo and Wildlife Research (IZW), Alfred-Kowalke-Strasse 17, 10315 Berlin, Germany; 3 Centre for Integrative Biodiversity Research (iDiv) Halle-Jena-Leipzig, Deutscher Platz 5e, 04103 Leipzig, Germany

## Abstract

Ecological disturbances are important drivers of biodiversity patterns. Many biodiversity studies rely on endpoint measurements instead of following the dynamics that lead to those outcomes and testing ecological drivers individually, often considering only a single trophic level. Manipulating multiple factors (biotic and abiotic) in controlled settings and measuring multiple descriptors of multi-trophic communities could enlighten our understanding of the context dependency of ecological disturbances. Using model microbial communities, we experimentally tested the effects of imposed disturbances (i.e. increased dilution simulating density-independent mortality as press or pulse disturbances coupled with resource deprivation) on bacterial abundance, diversity and community structure in the absence or presence of a protist predator. We monitored the communities immediately before and after imposing the disturbance and four days after resuming the pre-disturbance dilution regime to infer resistance and recovery properties. The results highlight that bacterial abundance, diversity and community composition were more affected by predation than by disturbance type, resource loss or the interaction of these factors. Predator abundance was strongly affected by the type of disturbance imposed, causing temporary relief of predation pressure. Importantly, prey community composition differed significantly at different phases, emphasizing that endpoint measurements are insufficient for understanding the recovery of communities.

## Introduction

Ecological disturbances affect interspecific interactions and, consequently, community dynamics^1,2^. Trophic interactions also play a crucial role in community dynamics as predators shape prey communities by affecting the strength of species interactions^3–13^. However, there is a lack of studies on the combined effects of disturbances and predators on prey communities^14^. Such studies would not only help to explain the response of complex multitrophic communities^15^ to anthropogenic disturbances, they are also indispensable for understanding synergistic and compensatory effects on the communities, which may cause ecological surprises and even irreversible outcomes^16^.

Disturbances are characterized by their intensity, frequency and extent, and are often classified as short-term and discrete (pulse disturbances) or long-term and continuous (press disturbances) events^17,18^. These disturbance characteristics largely determine the observed community responses, because they affect species reproduction and survival, as well as interactions between species^19^. For instance, press disturbances can influence community attributes such as relative species abundances beyond normal background variation, whereas pulse disturbances usually cause dramatic structural and functional community shifts^17^. As a result, communities and their functions may follow different trajectories during the disturbance and after the disturbance ends: *(i)* the community structure remains the same (resistance), *(ii)* the community structure changes, but over time returns to its original state (recovery), *(iii)* the community structure is changed but function is maintained (functional redundancy), *(iv)* function changes but the community structure does not change (functional plasticity) and *(v)* the community structure changes and neither returns nor maintains its function^20–22^. However, most of the studies do not quantify and compare the community composition between the pre-disturbance phase, the phase following the onset of the disturbance, and the phase after the disturbance (but see^10,21,23,24^).

When causing mortality, disturbances may alter niche structure and nutrient fluxes. For instance, fire results in biomass sequestration in soil and strong rainfall can transfer terrestrial nutrients to aquatic systems^25^. Such resource deprivations may occur in different disturbance scenarios. Prolonged severe drought may cause, for example, a gradual loss of water resources and progressive changes in the physiological status of plants^26^.

Partial deprivation of resources may also change the competition patterns between species, giving an advantage to species with high resource affinity^27^.

Predation and disturbances may interact in complex ways in their effects on prey communities^28^. Predation as a biotic pressure in concert with abiotic disturbances may change prey abundances, diversity and community composition^29^. Many predators are characterized by selective feeding/predation modes and predation success is controlled by traits of their potential prey, such as its size^30,31^. The predators’ physiological states, sizes and growth rates can be affected by abiotic disturbances; predators with slow growth rates and large body sizes are usually most affected by disturbances. As a result, reduced top-down control due to disturbances may even cause prey outbreaks^32^.

In this study, we addressed the combined impact of predation and disturbances on species abundances, diversity and community composition. To this end, we assembled communities of bacterial prey and a protist predator in controlled laboratory settings and monitored the community dynamics prior to disturbances, during disturbances and four days after disturbances (i.e. the return to experimental pre-disturbance conditions). More specifically, we coupled increased community dilution simulating mortality with gradual or instant resource deprivation. Measurements taken at different phases of the experiment allowed us to examine the resistance and recovery properties of the communities. Since the disturbances that we imposed remove equal fractions of prey and predator individuals, we expected that a reduced predator density (resulting in larger clearance zones for nourishment) might favor the prey due to reduced contact frequency^33–36^. This, in turn, would result in higher prey abundances. Thus, the magnitude of change in prey populations, their resistance or recovery upon disturbance might be mediated by the predator.

## Methods

### Experimental methods

#### Organisms

*Agrobacterium rhizogenes* (α-Proteobacteria), *Kocuria rhizophila* (Actinobacteria), *Sphingobium sp*. (α-Proteobacteria) and *Williamsia sp*. (Actinobacteria) were used as prey. These prey species are common free-living microorganisms in aquatic and soil ecosystems and vary in their population growth (Supplementary Material A) covering the breadth of population growths representative of natural communities^6,11,37^. Prior to the experiments, they were grown in pure cultures in Brunner-CR2 medium^6^ overnight at 25 °C in a shaking incubator. Abundance of prey individuals during the experiment was estimated with a particle counter (Multisizer™ 3, Coulter Counter, Beckman Coulter, USA). Precultures of the ciliate predator *Tetrahymena pyriformis* (with an average length and width of 20 × 50 µm) were maintained in proteose peptone yeast extract medium^38^ at 25 °C in an incubator without shaking. They were cultivated axenically (growth only on dissolved nutrients without bacteria) before the experiments to avoid transfer of unwanted bacteria to the experimental cultures. Prior to the experiment they were concentrated by centrifugation (10 minutes, 1,000 g) and washed with experimental medium. Controls without microorganisms, samples plated at each sampling point, and the previously known fingerprinting pattern of each species were used to screen for possible contamination. All strains are available on request from the public Culture Collection of the Department of Environmental Microbiology at the Helmholtz Centre for Environmental Research – UFZ (http://www.ufz.de/index.php?en=13354).

#### Experimental design

Static microcosms consisted of 20 mL of Brunner-CR2 medium in 50-mL cell culture flasks which were incubated at 25 °C in the dark. Prey cultures were diluted in the experimental medium evenly; total prey number was adjusted to 1.8 × 10^7^ cells mL^−1^, predator number was 4.2 × 10^4^ cells mL^−1^. This computes to approximately 400 prey per predator^6,10,11,37^. We performed daily 10-fold dilutions by transferring 10% of the community into a fresh medium thus eliminating the complications caused by dead cell debris, low oxygen levels and influences of high culture density. This replacement is necessary to prevent population collapse^39^. Cultures were shaken well before each transfer and sampling.

**Figure 1:**
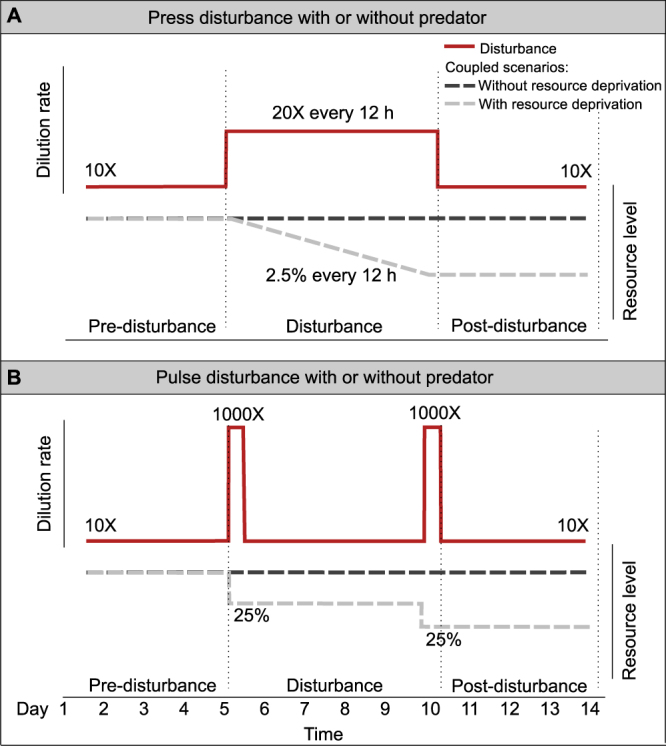
Experimental design. (**A**) Press disturbance with or without the predator. (**B**) Pulse disturbance with or without the predator. Dilution factors indicate the strength of simulated mortality and resource levels indicate the degree of resource deprivation: For press disturbance 2.5% reduction steps with 20-fold community dilution at each step, and, for pulse disturbance, 25% reduction steps with 1,000-fold community dilution at each step was applied. Vertical dashed lines delimit the three phases and indicate the times of sampling.

Thirty microcosms were started with the same inoculum and incubated for 14 days, corresponding to approximately 30–60 generations (Supplementary Material F) for the prey and the predator species used. We employed two trophic regimes (predator absent and present), two disturbance types (press and pulse), and two kinds of resource deprivation (absent and present). Resource deprivation was discrete in pulse disturbance experiments and gradual in the press disturbance experiment (Fig. 1). Each treatment was replicated three times and randomly placed in the incubator. We ran controls (without any disturbance) for 14 days as daily serial transfers involving 10-fold dilution throughout the experiment. The pre-disturbance regime consisted of four days of daily serial transfers involving 10-fold dilution to reach the equilibrium dynamics. Simulated press disturbance was then imposed as a 20-fold community dilution of the fresh medium every 12 hours over five days, whereas pulse disturbance was imposed as two 1,000-fold community dilutions within five days (on day 5 and 9; Fig. 1). Note that this simulated mortality is by definition different from mortality caused by disease, stress, intoxication, or predation, which leaves at least part of the dead biomass in the system.

In addition, we simulated resource deprivation by diluting the pre-disturbance medium that serves as a resource for the prey with autoclaved distilled water (Fig. 1). In press disturbance experiments, we diluted the resource gradually by 2.5% at each of the 20 disturbance transfers. In pulse disturbance experiments, we reduced the resource by 25% at each of the two disturbance transfers. These treatments finally resulted in a deprivation of 50% of the initial resources and remained at this resource level during the post-disturbance stage. Disturbances were followed by a period during which pre-disturbance dilution regimes (daily 10-fold dilution) were applied. Samples were taken prior to the disturbance (day 5), at the end of the disturbance (day 10) and four days after the disturbance (day 14).

#### Community composition estimation

Bacterial community composition was estimated by 16 S rRNA-gene based terminal restriction fragment length polymorphism (T-RFLP) analysis. Applying the restriction enzyme MspI made it possible to distinguish the specific T-RF of each bacterial prey species. Data were normalized to eliminate differences in total signal intensity between the different samples. We used only the four species-specific T-RFs and their relative abundances for the analyses. The T-RF of one species (*Williamsia sp*.) was absent due to competitive exclusion, and was thus removed from the analysis (see Supplementary Material B for a detailed description).

#### Species abundance estimation

Cell numbers of *Tetrahymena pyriformis* were estimated by counting cells fixed with 0.2% Lugol’s iodine solution under an inverted microscope (Olympus CKX-41) with the help of a counting chamber (Sedgewick Rafter Cell, Pyser-SGI Limited, UK). A subsample of the microbial community was fixed with 4% paraformaldehyde solution and total abundance of bacteria was estimated using a particle counter as mentioned above.

### Data analysis

All statistical analyses and visualizations were performed in R version 3.4.0^40^. We used *alpha* level 0.05 unless stated differently. All source codes used in this manuscript are available upon request. Our four dependent variables were total prey and predator abundance, prey diversity (measured using the Shannon-Weiner index) and community composition. We calculated the magnitude of change in total prey abundance relative to control treatments (averaged over replicates) without predation and disturbance and the magnitude of change in predator abundance relative to control treatments without disturbance: ${A}_{relative}=\,\mathrm{ln}({A}_{treatment})/\,\mathrm{ln}({\bar{A}}_{control})$. By doing so, we accounted for any directional change with time in control treatments. We assessed the impact of predation, disturbance and their interaction on prey abundances, and of disturbance on predator abundance with linear mixed effect models using the function mixed() in the afex package^41^. Main effects of predation, disturbance type, resource deprivation and phase were included in the analysis. Additionally, two-way interactions between all main effects were included. We did not include higher-order interactions to avoid overfitting. The phase was treated as a fixed factor, because we were specifically interested in the differences of response variables at the different phases. The model included a microcosm as a random intercept effect structure to account for variation among cultures due to factors other than those included as explanatory in the model. The significance of effects was tested using two-tailed Type III F- on the global model using a parametric bootstrap with 10,000 simulations. Model residuals were visually assessed for homogeneity and normality. The effects on predator abundance were assessed analogously by using disturbance type, resource deprivation and their interactions as fixed effects and individual microcosms as random effects.

Prey diversity was calculated with the Shannon-Weiner index (*H*)^42^. We calculated the magnitude of prey diversity change relative to the control (averaged over replicates) treatment as: ${H}_{relative}={H}_{treatment}/{\bar{H}}_{control}$ and used linear mixed effect models as described above. The results of the mixed-model analyses are presented as analysis of variance (ANOVA) tables (Tables 1–3). Pairwise differences between each treatment (predation, disturbance and resource deprivation) and at each time point were assessed with t-tests using the Satterthwaite approximations for denominator degrees of freedom using the lsmeans^43^ and multcompView packages^44^. Bonferroni-Holm corrections were used to take into account multiple comparisons. Model predictions were visualized with the sjPlot package^45^. Deviation coding approach was used, which compares the individual treatment means with the grand mean. We checked if inferences changed due to the averaging of the control replicates by pairing each of the treatment replicates with one of the randomly sampled control replicates and by subsequently calculating the respective relative abundance. The abundances of some species were so low that they resulted in relative abundances (*A*_*relative*_) that were too low. Similarly, we had some samples where only the most dominant species was detected by the fingerprinting method; this may have been caused by the reduced detection limit due to the resolution power of the method and the sample size. Since such extreme values may influence the model fit we examined Cook’s distances to assess the level of influence of extreme data points using the influence.ME package^46^. Influential extreme data points were removed. To assess how removal of extreme data points affected the inferences we also run analyses on the complete data set.

**Table 1:** Fixed effects in linear mixed-effects models of prey and predator abundance response to predation and disturbance. Df is degrees of freedom, χ^2^ and p values were derived from the parametric bootstrap. Significant effects are highlighted in bold.

*Total prey abundance*			
Effects	df	χ^2^	p
**Predation**	**1,71**	**134.35**	**<0.001**
**Disturbance**	**1,71**	**20.97**	**<0.001**
**Resource**	**1,71**	**19.43**	**<0.001**
**Phase**	**2,71**	**215.11**	**<0.001**
Predation x disturbance	1,71	0.56	ns.
**Predation x resource**	**1,71**	**10.52**	**0.004**
Disturbance x resource	1,71	0.61	ns.
**Predation x phase**	**2,71**	**239.05**	**<0.001**
**Resource x phase**	**2,71**	**33.69**	**<0.001**
**Disturbance x phase**	**2,71**	**18.55**	**<0.001**
***Total predator abundance***			
**Disturbance**	**1,35**	**19.17**	**0.002**
Resource	1,35	3.21	ns.
**Phase**	**2,35**	**173.71**	**<0.001**
Disturbance x resource	1,35	1.67	ns.
**Disturbance x phase**	**2,35**	**48.21**	**<0.001**
**Resource x phase**	**2,35**	**31.49**	**<0.001**

We tested the impact of main effects and their interactions on the community composition using the relative prey species abundances with a redundancy analysis (RDA) using the rda() function in the vegan package^47^. Since the control communities did not differ significantly between the phases (F_2,9_ = F = 0.849, p = 0.451), we eliminated them from the analysis in order to test the effect of resource deprivation. We tested whether adding a given variable in presence of others would increase the amount of variation significantly by checking variance inflation factors (all variance inflation factors <10).

The data were assigned to subsets according to the three phases to assess how the communities were affected by the treatments between the different time frames. Accordingly, we compared the change in community structure from the pre-disturbance to the disturbance phase, from the disturbance to post-disturbance phase, and from the pre-disturbance to the post-disturbance phase. We performed PERMANOVA to test the significance of the change in community structure by using a full randomization test (9999 permutations) to calculate the F-statistics. Since sampling times are far enough apart compared to the generation times of the organisms, we assumed that temporal autocorrelation between repeated measurements is negligible. Our analysis further indicated the lack of time dependency in the data (Supplementary information G). Finally, we partitioned the percentage of variation explained by predation, disturbance treatments and their two-way interactions using RDA. We measured the variation in the levels of dispersion across treatments using the betadisper() function in the vegan package.

### Data availability

The datasets generated and/or analyzed during the current study are available from the corresponding author upon reasonable request.

## Results

### Effect of predation and abiotic disturbances on total species abundances

**Figure 2:**
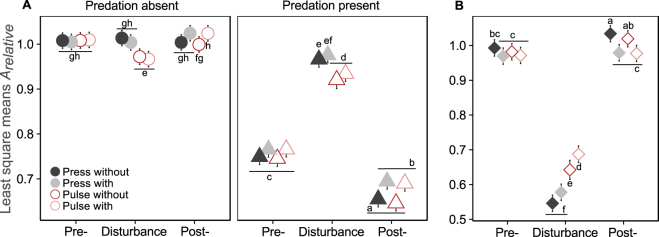
Magnitude of change in prey abundance relative to the control under the absence and presence of the predator and disturbance treatments (**A**), magnitude of change of predator abundance relative to the control under disturbance treatments (**B**). Colors code for the disturbance treatments; “press” and “pulse” are the disturbance types, “with” indicates that the disturbance is coupled with a resource deprivation and “without” is without resource deprivation. Points represent least square means for each treatment and the error bars are the confidence intervals. Bonferroni-Holm corrected multiple comparisons are shown as letters. Groups sharing the same letter are not statistically different.

We found significant effects of predation, disturbance type, resource deprivation and disturbance phase on the abundances of prey species (Table 1, Fig. 2A). Additionally, several interactions among the main effects were significant (Table 1).

Prey abundance was unaffected by resource deprivation when the predator was absent; however, in the presence of a predator prey abundance was higher under resource deprivation than with unchanged resources (Fig. 2A, Supplementary Fig. S1). In microcosms without a predator, prey abundance remained stable throughout all phases. In presence of a predator, on the other hand, prey abundance was lower in the pre-disturbance phase and increased during disturbance to an abundance similar to that observed without predation. In the post-disturbance phase, prey abundance dropped below pre-disturbance levels (Fig. 2A, Supplementary Fig. S1).

Predator abundance was affected by disturbance type, disturbance phase and several interactions between the main factors (Table 1). Predator abundances did not differ between press and pulse disturbance in the pre- and post-disturbance phases. However, during the disturbance phase the predator abundance was higher in pulse disturbance treatments than in the press disturbance ones (Fig. 2B, Supplementary Fig. S2). The impact of resource deprivation was only visible during the post-disturbance phase, that is, predator abundance was higher in the absence of resource deprivation.

### Effect of predation and abiotic disturbances on prey diversity

**Figure 3:**
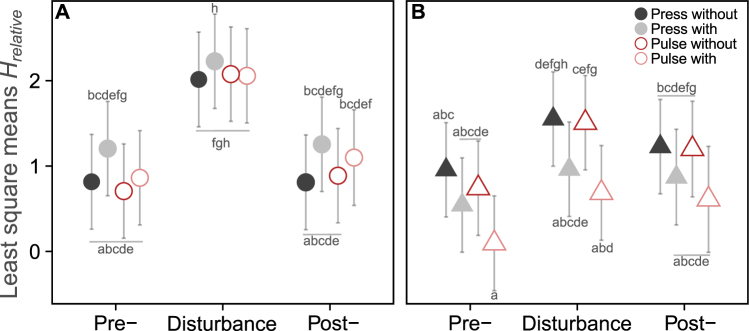
Magnitude of change in prey diversity *(H)* relative to the control under different disturbance treatments in the absence (**A**) or presence (**B**) of a predator. Colors code for the disturbance treatments, “press” and “pulse” are the disturbance types, “with” indicates that the disturbance is coupled with a resource deprivation and “without” is without resource deprivation. Points represent least square means for each treatment and the error bars are the 95% confidence intervals. Bonferroni-Holm corrected multiple comparisons are shown as letters. Groups sharing the same letter are not detectably different.

We found that predation and disturbance phase significantly affected prey diversity. We additionally found significant interaction between predation and resource deprivation treatment (Table 2). Thus, there was no difference in prey diversity when disturbance was coupled with resource deprivation in microcosms without a predator. However, in the presence of a predator resource deprivation had a negative effect on prey diversity (Fig. 3, Supplementary Fig. S3). The effect of predation on prey diversity changed over time: predation pressure reduced prey diversity in the pre-disturbance phase compared to the treatment without a predator. This effect was even more pronounced in the disturbance phase and diminished in the post-disturbance phase (Fig. 3, Supplementary Fig. S3). Nevertheless, prey diversity was slightly higher during the post-disturbance phase than during the pre-disturbance phase, both in the absence and in the presence of predation.

**Table 2:** Fixed effects in a linear mixed-effects model of prey diversity *(H)* response to predation and disturbance. Df is degrees of freedom, χ^2^ and p values were derived from the parametric bootstrap. Significant effects are highlighted in bold.

Effects	df	χ^2^	P
**Predation**	**1,71**	**17.66**	**0.002**
Disturbance	1,71	3.86	ns.
Resource	1,71	3.41	ns.
**Phase**	**2,71**	**49.23**	**<0.001**
Predation x disturbance	1,71	0.35	ns.
**Predation x resource**	**1,71**	**16.34**	**<0.001**
Disturbance x resource	1,71	1.65	ns.
**Predation x phase**	**2,71**	**14.98**	**0.004**
Disturbance x phase	2,71	0.89	ns.
Resource x phase	2,71	1.20	ns.

Differences in the individual means of each treatment from the mean over all treatments for all the linear mixed effect models are shown in Supplementary Figs S4–6 The results were qualitatively the same when we randomly coupled a control replicate with the treatment replicate for calculating the relative abundances/prey diversity and used the full dataset in analyses (no removal of extreme data points), see Supplementary Table S2–4

### Effect of predation and abiotic disturbances on overall prey community composition

**Figure 4:**
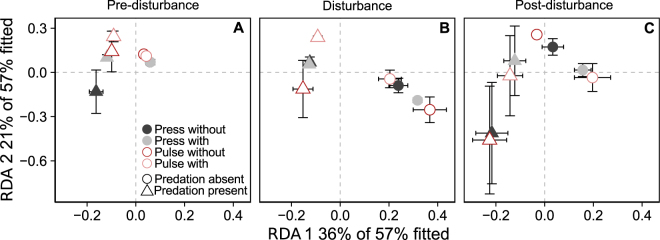
The first two axes of the RDA analysis. Circles represent the treatments without and triangles with predation. “Press” and “Pulse” are the disturbance types, “with” indicates that the disturbance is coupled with a resource deprivation and “without” is without resource deprivation. Error bars for vertical and horizontal axes display the ± standard error. Both axes are significant (RDA1: F_1,72_ = 57.415, p = < 0.001; RDA2: F_1,72_ = 33.202, p = < 0.001).

Prey community composition differed significantly between the different phases. Additionally, predator presence significantly affected prey community composition, and differently so in different phases (Table 3, Fig. 4, Supplementary Figs S7–8).

**Table 3:** Results of redundancy analysis. Df is degrees of freedom, F-statistics and p-values were derived from permutation tests. Significant effects are highlighted in bold and marginally significant effects (p < 0.1) in italics.

Effect	df	*Pre- vs. Disturbance*		*Disturbance vs. Post-*		*Pre- vs. Post-*	
		F	p	F	p	F	p
Predation	1,48	**72.506**	**<0.001**	**24.613**	**<0.001**	**24.613**	**<0.001**
Disturbance	1,48	1.875	ns.	0.038	ns.	0.038	ns.
Resource	1,48	**4.403**	**0.022**	*2.511*	*0.084*	*2.511*	*0.088*
Phase	1,48	**18.943**	**<0.001**	**3.272**	**0.043**	3.278	**0.042**
Predation × disturbance	1,48	1.079	ns.	0.051	ns.	0.051	ns.
Predation × resource	1,48	*2.895*	*0.071*	*5.009*	*0.006*	**5.009s**	**0.006**
Disturbance × resource	1,48	*2.647*	*0.087*	0.482	ns.	0.482	ns.
Predation × phase	1,48	**17.132**	**<0.001**	**9.262**	**<0.001**	**9.262**	**<0.001**
Disturbance × phase	1,48	2.098	ns.	0.038	ns.	0.038	ns.
Resource × phase	1,48	0.217	ns.	1.045	ns.	1.045	ns.

There was greater variability in the composition of communities exposed to the predator than those not exposed to a predator, as indicated by the large data spread of the respective replicate microcosms during pre- and post-disturbance (F_1/24_ = 8.657, p = 0.002; post-disturbance F_1/24_ = 11.262, p = 0.001 respectively). Resource deprivation was only significant in the pre-disturbance/disturbance comparison, while interaction between predation and resource deprivation gained importance in the disturbance/post-disturbance comparison (Table 3). Other marginal effects are shown in Table 3. In each comparison, the highest variation in community composition was explained by the effect of predation (Supplementary Fig. S9).

## Discussion

Understanding combined effects of multiple disturbances on microbial communities is essential in the face of ongoing global change and multiple disturbances acting simultaneously. Here we showed that prey abundance, diversity and community composition were more strongly affected by predation than by disturbances (resource deprivation and dilution). At the same time, the type of disturbance (pulse vs. press) had a strong impact on the abundance of the predator. Our experimental system is simplified and the results are not meant to be extended to complex microbial communities. Due to the low number of species involved, our inferences cannot be extrapolated to real-world ecosystems. The results obtained from this study do, however, provide a good basis for further studies.

In our experimental system the top-down control affected the prey abundances and community composition much more strongly than the bottom-up effects (Figs 2 and 4, Tables 1 and 3). Indeed, as hypothesized, the delayed recovery of the predator in the disturbance phase and thus a reduced top-down control resulted in prey abundances similar to treatments without predation pressure (Fig. 2, Table 1, Supplementary Fig. S1). These findings are in line with other studies showing that systems experiencing continuous or discrete disturbances imposed on predators (e.g. by hunting or anthropogenic removal) often show prey release^32,48^. For instance, hydrological disturbances in wetlands resulted in smaller predator size, which in turn led to excessive growth of prey^32^. Furthermore, in a microcosm study predators were found to reduce prey abundance by almost 50%, although disturbances diminished this effect significantly^49^.

Even though the effects of predation on prey abundances and community were the most pronounced, they were moderated by the bottom-up effects, underlining the importance of both abiotic and biotic factors for community dynamics^50^. Thus, the bottom-up effect in the form of a resource deprivation resulted in slightly higher prey abundance in the post-disturbance phase (Fig. 2A, Table 1, Supplementary Fig. S1). Such higher prey abundances under resource deprivation contradict the hypothesized positive effect of resource availability on the recovery of communities^27^. However, the observed effect is minor (compared to the effect of the predation) and may potentially be explained by the strong interspecific competition triggered by reduced resource availability. Such interspecific competition has indeed resulted in an increased relative abundance of the most competitive species *A. rhizogenes* (Supplementary information B and Table S1, Supplementary Fig. S7).

It is important to note that our results must be interpreted bearing in mind the caveats associated with our not fully factorial experimental design. For instance, different resource deprivation treatments were coupled with the two disturbance types, respectively. Although this design has limitations for understanding how resource deprivation and disturbance interact, the rationale behind it was to mimic plausible natural conditions, such as continuous resource removal due to periodic drought^51^ or rapid removal of biomass after flood events^52^.

Predator abundance decreased more strongly under press than under pulse disturbance. Each press disturbance event was applied every 12 h, which is closer to the generation time of the prey (ca. 2–4 generations per day) than the intervals between the two pulse disturbance events (5 d); press disturbance therefore has a higher potential to push populations to extinction or to an alternative state (e.g. equilibrium at lower population sizes)^53^. This effect, however, was only visible in the disturbance phase possibly indicating a high recovery potential of the predator independent of the disturbance type. Further, resource deprivation affected predator abundance positively during disturbance and negatively thereafter (Fig. 2B, Table 2, Supplementary Fig. S2). Such reduced predator abundance partly explains the higher prey abundance under those treatments. In short, mean prey and predator abundances tended to recover; still, in presence of a predator the abundance of prey was lower at the end of the experiment than in the pre-disturbance phase. This might be a transient behavior^54^ due to the short duration of the experiment.

In our experiment both predator and prey species were affected by disturbances at the same rate, however, the low population size of the predator was enough to initiate a prey release. A reduced abundance of individuals at the top trophic level is highly relevant, since a prolonged recovery time caused by an increased disturbance duration and strength, and diminished availability of alternative resources^10^ during which population size is low, poses an increased extinction risk^55^. Extinction of top predators may cause radical changes in ecosystems by altering community structure^8,33,56^. Specific prey groups may also increase in abundance and reduce the evenness of the community^57^, and even cause invasions^58^.

Note that *Tetrahymena* species are able to grow on dissolved carbon sources^59^ and foraging on bacteria may be flexible due to specific predator traits such as absolute time or effort needed for grazing and relative intake rates^60^. We ignored the consumption rate of the prey resources by the predator, because our previously conducted experiments showed it to be negligible (unpublished data). However, we do not exclude the possibility that the ability of the predator to feed on prey resources could have resulted in competition for the resources, which might have affected the response of the predator^10^.

Disturbance may affect diversity by enhancing coexistence and evenness in communities^61^. In our experiments, in contrast, prey diversity was lower in the presence of the predator. Interaction of predation with resource deprivation also correlated with a reduced diversity, which might result from the higher relative abundance of dominant species in the community (Fig. 3, Table 2, Supplementary Fig. S7). Both predation and resource deprivation acted as ‘environmental filters’ according to the niche principle (*sensu*^62^) by filtering out the species that cannot sustain a certain level of predation or resource limitation, thus resulting in the replacement of those species by the resistant and competitive ones; accordingly, the community structure became more simplified.

Previous studies found that ecosystem functions are usually affected by disturbances depending on their intensities and frequencies^53,63^. For instance, recovery potential after a pulse disturbance (e.g. flood) might be high, whereas recovery after a press disturbance (e.g. drought) may take considerable time^18^. Yet, the disturbance type had a small or no consistent influence on the prey communities in our study. We found that predation explained most of the variation in the community composition (Fig. 4, Table 3, Supplementary Fig. S9). It is possible that the difference between press and pulse disturbances in our experiments was not strong enough to cause a change in the community structure.

Our study demonstrates importance of following the prey community over time, because both prey abundance and community composition changed during and after disturbance^64^. Moreover, the effect of disturbance types and resource deprivation on the prey community was phase-dependent (Figs 2–4). However, our work can only offer a limited understanding of the temporal community dynamics because we sampled the community composition only once after the disturbance, at a time point that may have been too early to infer full recovery. Indeed, most of the literature suggests that microbial communities recover to their original state quickly^20^. However, at first sight, our communities may seem to be still in a transient state^65^ due to the short duration of the experiments, that is, given more time, they might turn to the original state. Yet, under predation pressure communities have ultimately changed (i.e., we observed species replacement) and a return to a pre-disturbance composition did not seem possible within the time-frame of the experiment (Supplementary Fig. S7). Similarly to a previous study^49^, in control communities (with predation, without disturbance) dominant species were replaced by a resistant one. This resulted in a clear distinction between communities with and without disturbances under predation (Supplementary Fig. S7–8), indicating a possible trade-off between competitive ability and resistance to predation^66,67^. Note that such community change may be a result of our experimental setup. In particular, we removed 90% of the populations during each transfer, thus, the risk of stochastic extinction might be elevated. Additionally, our experimental system was closed and did not allow immigration into the microcosms, which is known to maintain local biodiversity^68–70^. Several studies have also shown that rapid prey adaptation within generations is possible^71,72^ and environmental fluctuations may intervene in adaptation processes^73^.

To sum up, we found that the interplay between predation and disturbance determined the response of the bacterial community in terms of diversity and structure. We demonstrated that it is essential to consider multiple response measures from species abundances to community structure, because they differ in their sensitivity to disturbances, as reflected by different recovery dynamics. Future studies should include measurements of community composition at several time points (e.g. see ref.^24^) throughout the disturbance exposure to understand community stability properties and mechanisms underlying them. We found that even for such a relatively simple two-trophic level community the responses to multiple abiotic and biotic disturbances were complex and in several cases disturbances interacted in their effects on bacterial community. However, predation was the main driver of prey abundance and community composition, indicating that a significant portion of the variation in prey community response is due to the top-down control, which deserves further attention in future disturbance ecology research.

## Electronic supplementary material

Supplementary Information
