# Supplementary material for: Interactions between predation and disturbances shape prey communities
Source: Sci Rep. 2018 Feb 14;8:2968. doi: 10.1038/s41598-018-21219-x (PMC5813231; doi:10.1038/s41598-018-21219-x)
Supplement: Supplementary file 1 — Supplementary Information [file 41598_2018_21219_MOESM1_ESM.pdf]

Supplementary Information 1

Interactions between predation and disturbances shape prey communities

Canan Karakoç[1], Viktoriia Radchuk[2], Hauke Harms[1,3], Antonis Chatzinotas[1,3*]

[1]Department of Environmental Microbiology, Helmholtz Centre for Environmental Research - UFZ, Permoserstrasse 15, 04318 Leipzig, Germany

[2]Department Evolutionary Ecology, Leibniz Institute for Zoo and Wildlife Research (IZW), Alfred-Kowalke-Strasse 17, 10315 Berlin Germany

[3]Centre for Integrative Biodiversity Research (iDiv) Halle-Jena-Leipzig, Deutscher Platz 5e, 04103 Leipzig, Germany

Corresponding author: Antonis Chatzinotas, antonis.chatzinotas@ufz.de

A. Extended description of the prey relative abundance determination methods

Bacterial DNA was extracted using the NucleoSpin® Tissue Kit (Macherey-Nagel, Düren, Germany) and 16S rRNA genes were amplified by PCR using the FAM-labelled bacterial forward primer 27f (5-AGA GTT TGA TCM TGG CTC AG-3) and the reverse universal primer 1492r (5-CGG TTA CCT TGT TAC GAC TT-3). Each 25-mL reaction consisted of 12,5 µl master mix (Bioline, London, UK), 5pmol of each primer and 10ng of sample DNA. Thermocycling was carried out with an initial denaturation step at 95°C (5 min), 35 cycles of denaturation at 95°C (45 sec), annealing at 56°C (45 sec) and primer extension at 72°C (1 min) and extension step at 72°C (10 min). Fluorescently labeled PCR products were purified with SureClean (Bioline, London, UK) and approximately 20 ng of them were digested with2U restriction enzyme MspI (New England Biolabs GmbH, Frankfurt/Main, Germany) at 37°C during 16 hours. Terminal-restriction fragments (T-RFs) were separated and detected in a capillary sequencer (ABI Prism® 3130, Applied Biosystems, Foster City, CA, USA) with an internal size standard (GeneScan 500 ROX, Applied Biosystems). T-RFLP electropherograms were analyzed using GeneMarker® (Soft Genetics, State College, PA, USA).

B. Competitive ability of prey species used

To determine the competitive ability we cultivated the prey organisms for 24 h alone and in all possible pairwise combinations, starting with the same cell number. We plated a subsample and counted the colony forming units (cfu) of each organism using their distinguishable colors after 2 days [1]. All monocultures and combinations were replicated four times. We estimated competitive ability of species following the approach of Fox (2002) and Haddad et al. (2008). We first calculated the response of species *i*, and the effect of *i* on

j when they grew together using the following index [2]:

$$CR_{ijk} = {(K_i - N_{ijk})}/{K_i} \quad (1)$$

where K_i is the abundance of species *i* in the single species trial, averaged over the four replicates, and N_{ijk} is the abundance of species *i* when grown together with competitor *j* in replicate *k*.

We then calculated the competitive ability considering sum of all the responses and effects of a species [3]:

$$CA_j = \frac{1}{n}\left(\sum_i CR_{ij} - \sum_i CR_{ji}\right) \quad (2)$$

where the first summation is the effect of a focal species on each other species, the second summation is the response of a focal species, and *n* is the number of species. Negative *CA* indicates poor competitive ability, whereas positive C indicates good competitive ability. Zero *CA* indicates that the effects of a focal species on each other species are cancelled out by the responses of the focal species to other species, resulting in a neutral net competitive ability of the species.

Table S1. Competitive ability of the species. Mean competitive ability and confidence intervals were determined by bootstrapping procedure.

Species	Ability	Percentiles
Agrobacterium sp.	0,487	(0,426; 0,530)
Koccuria sp.	0,292	(0,271; 0,330)
Sphingobium sp.	0,041	(0,006; 0,087)
Williamsia sp.	-0,820	(-0,851; -0,789)

C. Predictions of linear mixed effect models

Figure S1. Significant two-way interactions in the model predicting relative prey abundance. Shaded points are all the predictions, solid points are the means and vertical lines are the confidence intervals.

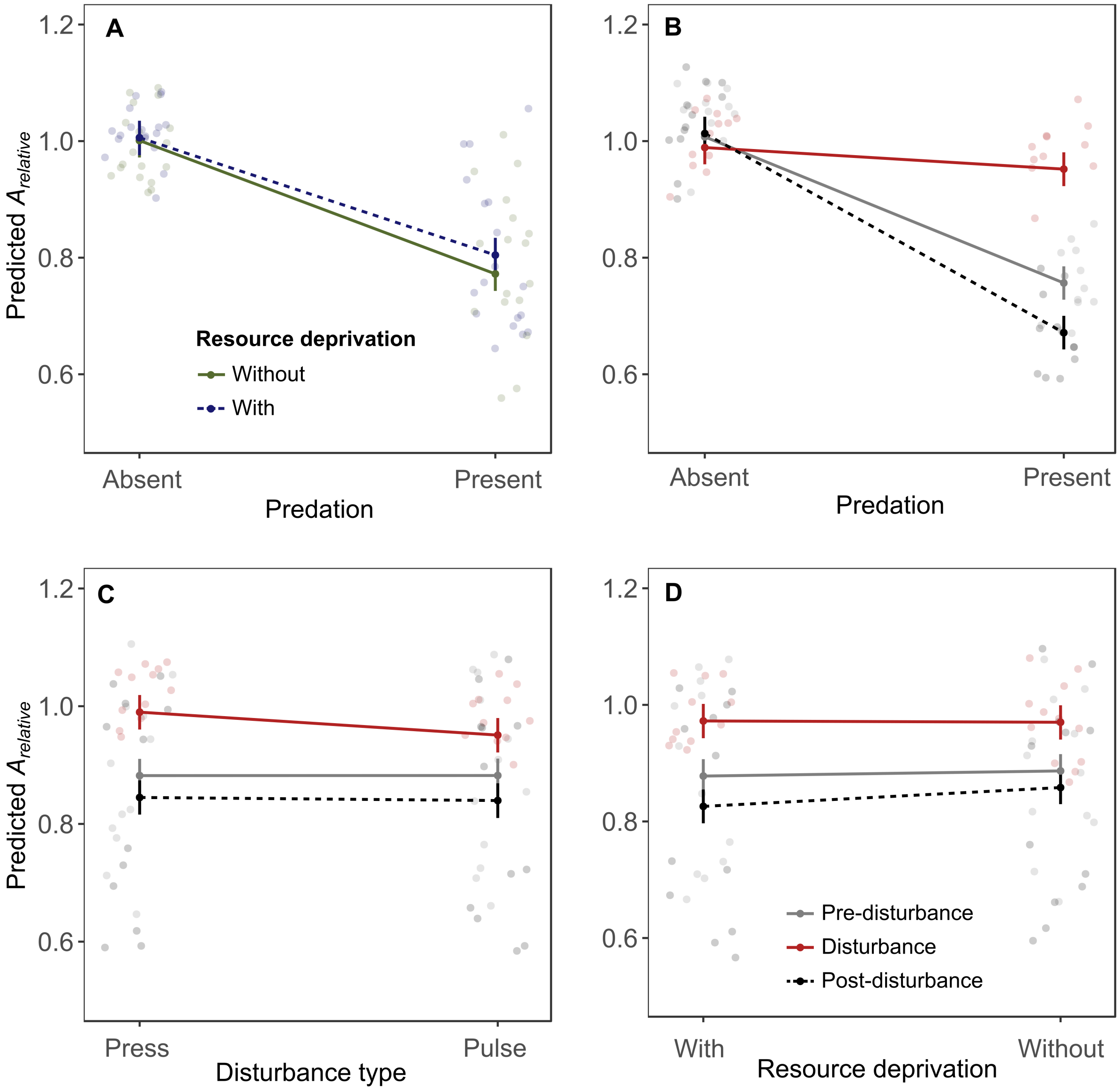

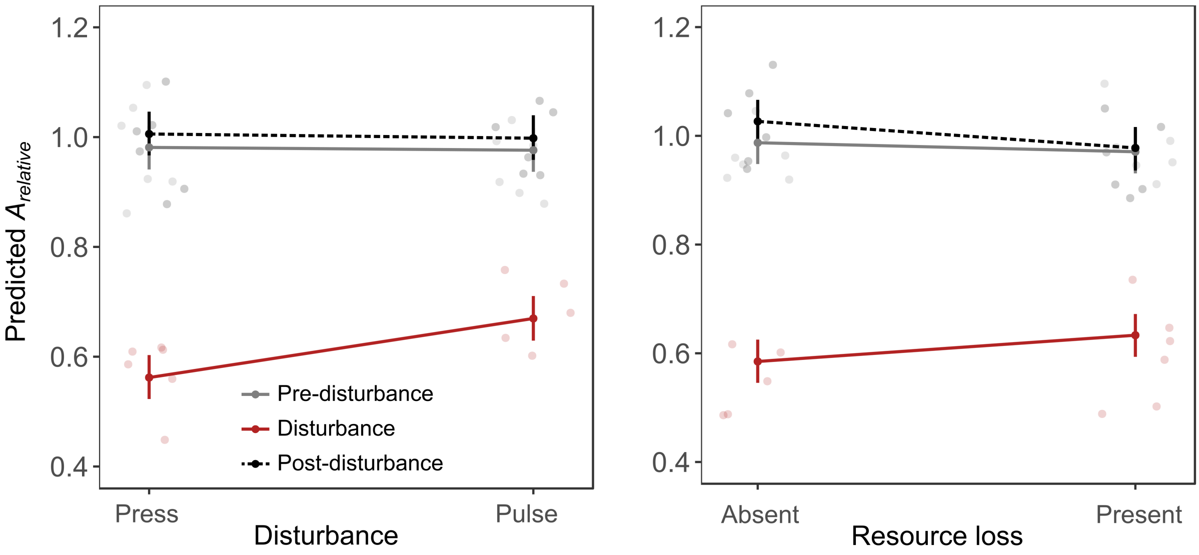

Figure S2. Significant two-way interactions in the model predicting relative predator abundance. Shaded points are all the predictions, solid points are the mean and vertical lines are the confidence intervals.

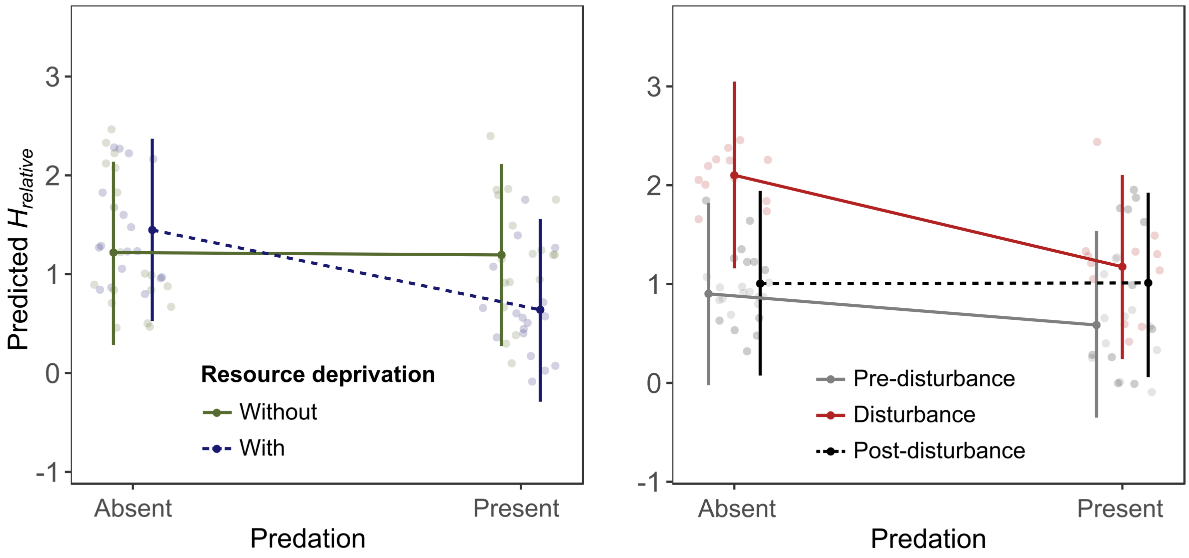

Figure S3. Significant two-way interactions in the model predicting relative prey diversity. Shaded points are all the predictions, solid points are the mean and vertical lines are the confidence intervals.

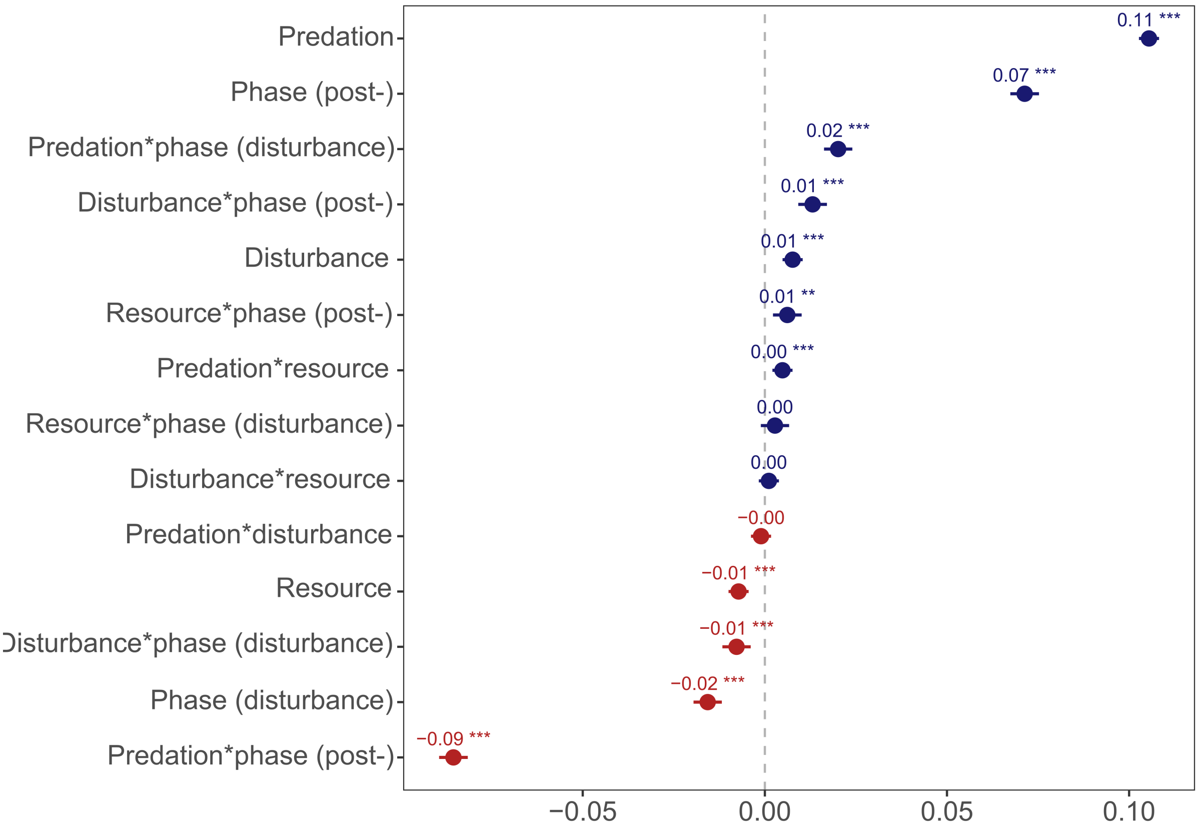

Figure S4. Regression coefficients from linear mixed effect model of effect of experimental treatments on relative prey abundance. Baseline is the mean of all factors. Shown factor levels are predation (present), disturbance type (pulse) and resource deprivation (present). Disturbance phases were stated in brackets. Points represent the mean and lines are the 95% confidence intervals. Positive and negative effects are shown as blue and red respectively (Significance codes: 0 '***' 0.001 '**' 0.01 '*' 0.05 '.' 0.1 ' ' 1).

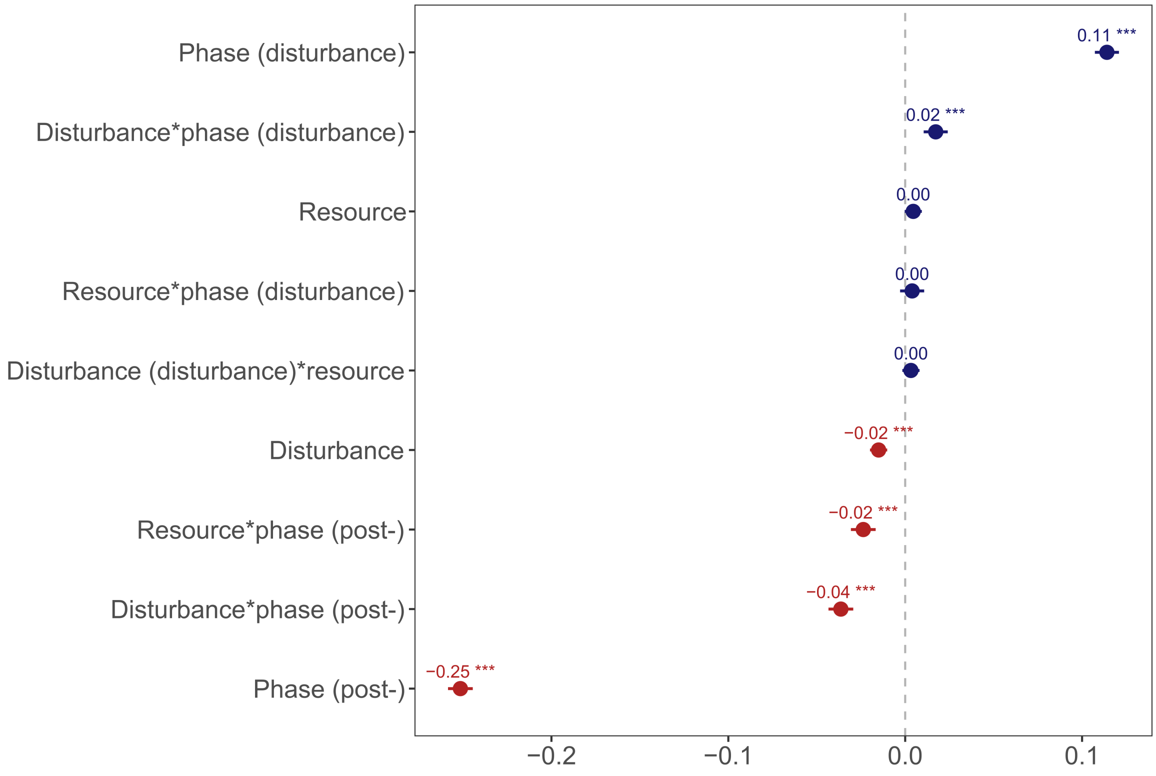

Figure S5. Regression coefficients from linear mixed effect model of effect of experimental treatments on relative predator abundance. Baseline is the mean of all factors. Shown factor levels are predation (present), disturbance type (pulse) and resource deprivation (present). Disturbance phases were stated in brackets. Points represent the mean and lines are the 95% confidence intervals. Positive and negative effects are shown as blue and red respectively (Significance codes: 0 ‘***’ 0.001 ‘**’ 0.01 ‘*’ 0.05 ‘.’ 0.1 ‘ ’ 1).

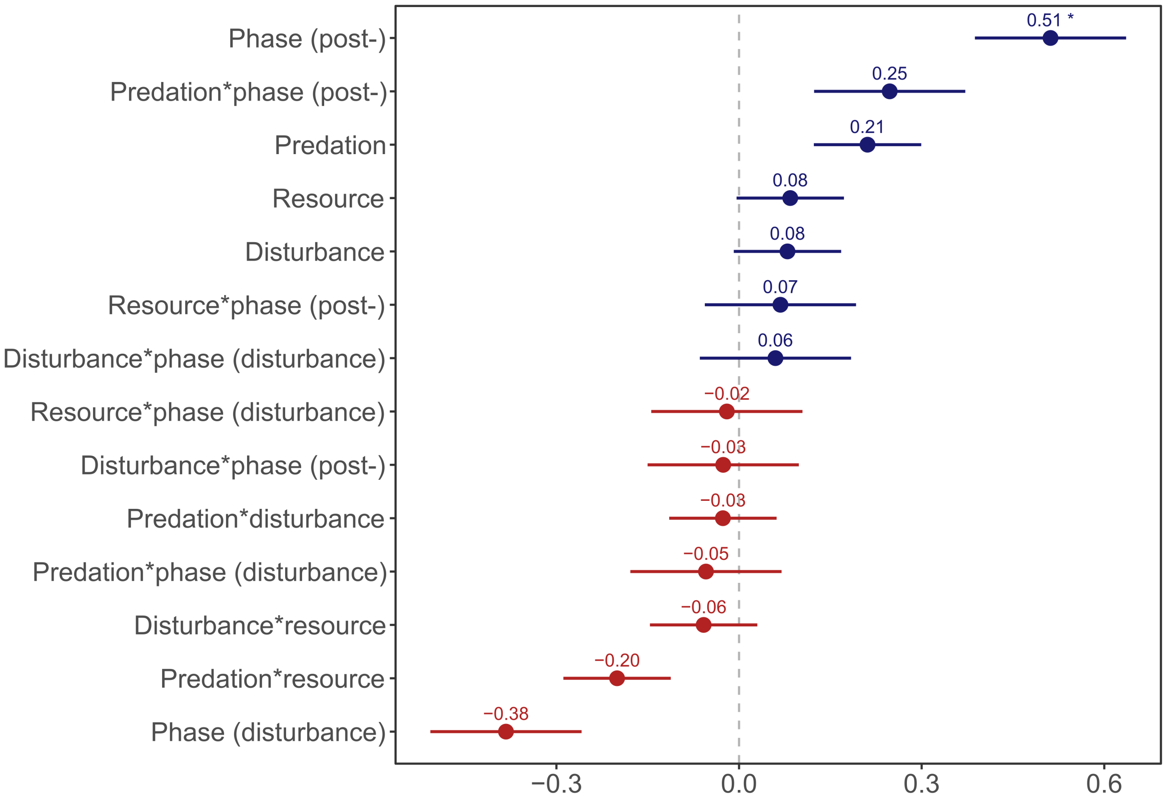

Figure S6. Regression coefficients from linear mixed effect model of effect of experimental treatments on relative prey diversity. Baseline is the mean of all factors. Shown factor levels are predation (present), disturbance type (pulse) and resource deprivation (present). Disturbance phases were stated in brackets. Points represent the mean and lines are the 95% confidence intervals. Positive and negative effects are shows as blue and red respectively (Significance codes: 0 '***' 0.001 '**' 0.01 '*' 0.05 '.' 0.1 ' ' 1).

D. Relative abundances and community structure of prey species in different treatments

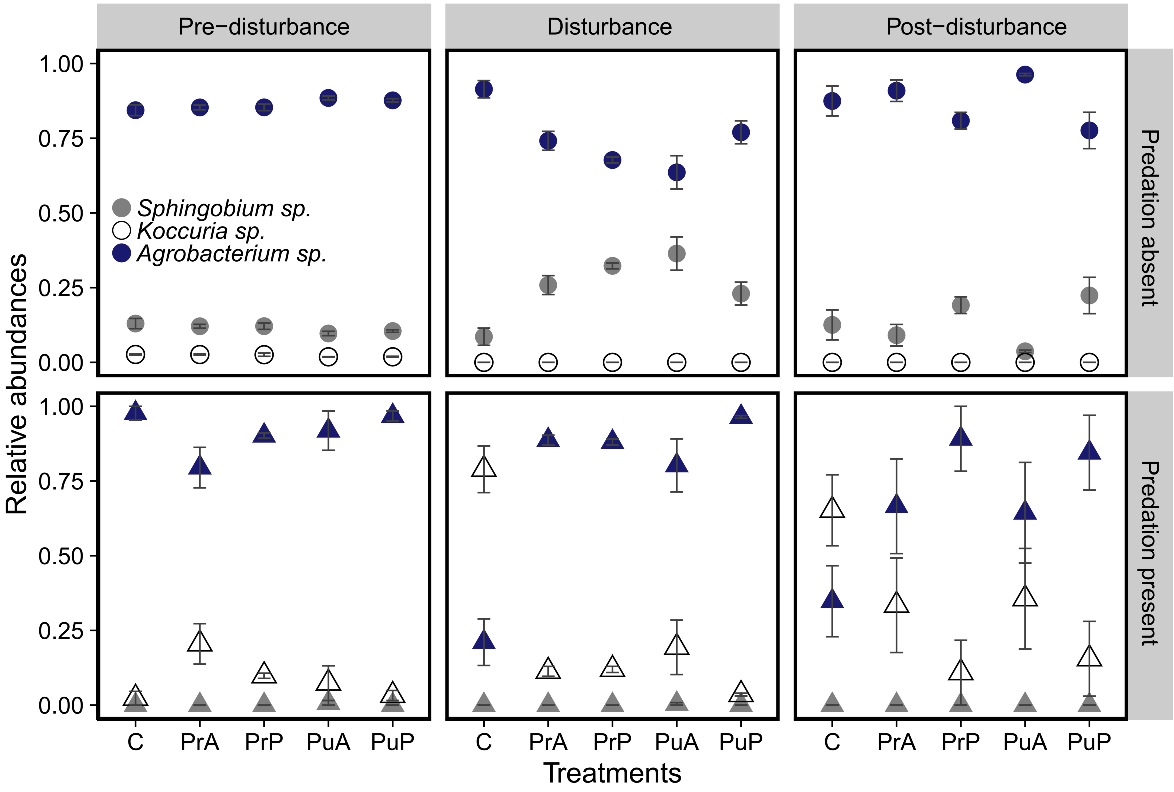

Figure S7: Relative abundances of the prey species determined by T-RFLP profile as explained in the main text. C: Control, Pr: Press disturbance, Pu: Pulse disturbance, A: Without resource deprivation, P: With resource deprivation.

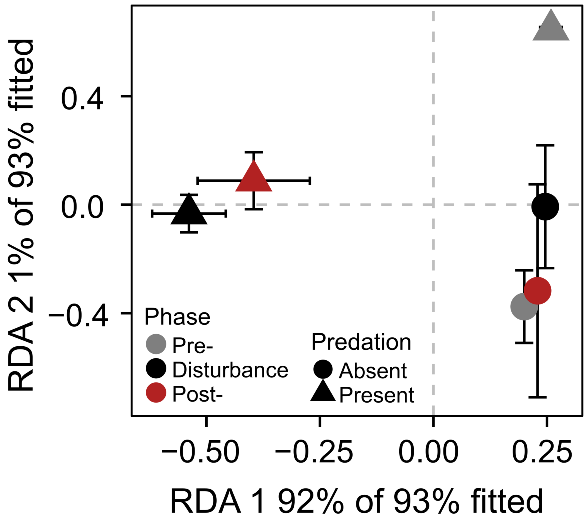

Figure S8. Communities without disturbance and resource deprivation treatments. The first two axes of the RDA analysis. Circles represent the control treatments without and triangles with predation. Colors code for the disturbance phases. Error bars display the ± standard error for vertical and horizontal axes.

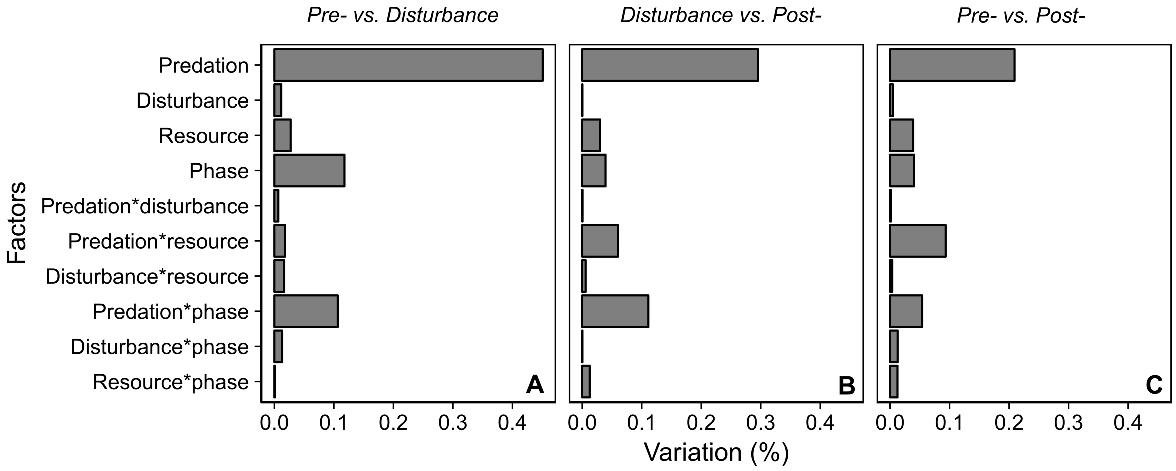

Figure S9. Variation in community composition explained by the main factors and their two-way interactions for the pre- vs. disturbance **(A)**, disturbance vs. post- **(B)** and pre- vs. post-disturbance phase **(C)**. Total variation explained for different phase comparisons is 67%, 56% and 47%.

E. Sensitivity of results to data removal and averaging of the control replicates

Table S2. Fixed effects in linear mixed-effects models of prey and predator abundance response to predation and disturbance on the full data set. Df is degrees of freedom, χ^2 and p values were derived from parametric bootstrap. Significant effects are highlighted in bold.

Total prey abundance			
**Effects**	**df**	χ^2	**p**
**Predation**	**1,72**	**134.35**	**<0.001**
**Disturbance**	**1,72**	**20.97**	**<0.001**
**Resource**	**1,72**	**19.43**	**<0.001**
**Phase**	**2,72**	**215.11**	**<0.001**
Predation x disturbance	1,72	0.56	ns.
**Predation x resource**	**1,72**	**10.52**	**0.006**
Disturbance x resource	1,72	0.61	ns.
**Predation x phase**	**2,72**	**239.05**	**<0.001**
**Resource x phase**	**2,72**	**33.69**	**<0.001**
**Disturbance x phase**	**2,72**	**18.55**	**0.006**
Total predator abundance			
**Disturbance**	**1,36**	**14.33**	**0.002**
Resource	1,36	3.96	ns.
**Phase**	**2,36**	**155.20**	**<0.001**
Disturbance x resource	1,36	0.02	ns.
**Disturbance x phase**	**2,36**	**41.18**	**<0.001**
**Resource x phase**	**2,36**	**14.55**	**0.006**

Table S3: Fixed effects in linear mixed-effects model of prey diversity *(H)* response to predation and disturbance on the full data set. Df is degrees of freedom, χ^2 and p values were derived from the parametric bootstrap. Significant effects are highlighted in bold.

**Effects**	**df**	χ^2	**P**
**Predation**	**1,72**	**14.14**	**0.01**
Disturbance	1,72	1.70	ns.
Resource	1,72	1.96	ns.
**Phase**	**2,72**	**44.81**	**<0.001**
Predation x disturbance	1,72	0.03	ns.
**Predation x resource**	**1,72**	**12.93**	**<0.001**
Disturbance x resource	1,72	0.73	ns.
**Predation x phase**	**2,72**	**16.60**	**0.001**
Disturbance x phase	2,72	1.63	ns.
Resource x phase	2,72	2.13	ns.

Table S4. Effects of the main factors and interactions on the abundances/diversity relative to the averaged and randomly sampled control replicate for each treatment. Significance codes: 0 '***' 0.001 '**' 0.01 '*' 0.05.

Prey abundance relative to the control treatment		
Effect	***Randomly Sampled***	***Averaged***
Predation	***	***
Disturbance	***	***
Resource	***	***
Phase	***	***
PredationXdisturbance	ns.	ns.
PredationXresource	**	**
DisturbanceXresource	ns.	ns.
PredationXphase	***	***
DisturbanceXphase	***	***
ResourceXphase	**	***
Predator abundance relative to the control treatment		
Effect	***Randomly Sampled***	***Averaged***
Disturbance	***	**
Resource	ns.	ns.
Phase	***	***
DisturbanceXresource	ns.	ns.
DisturbanceXphase	***	***
ResourceXphase	***	***
Prey diversity relative to the control treatment		
Effect	***Randomly Sampled***	***Averaged***
Predation	*	**
Disturbance	ns.	ns.
Resource	ns.	ns.
Phase	***	***
Predation*disturbance	ns.	ns.
Predation*resource	***	***
Disturbance*resource	ns.	ns.
Predation*phase	**	**
Disturbance*phase	ns.	ns.
Resource*phase	ns.	ns.

F. Estimation of generation times of species

In order to have an idea about the life history of the used bacterial species, we monitored the growth curves of pure cultures (n=8) in 24-well plates at the experimental temperature and in the experimental medium. Optical density was measured at 600nm every 30min. We then fitted a logistic growth model using nls function in R. The used species had approximately 2-4 generations per day (Figure S10, Table S5).

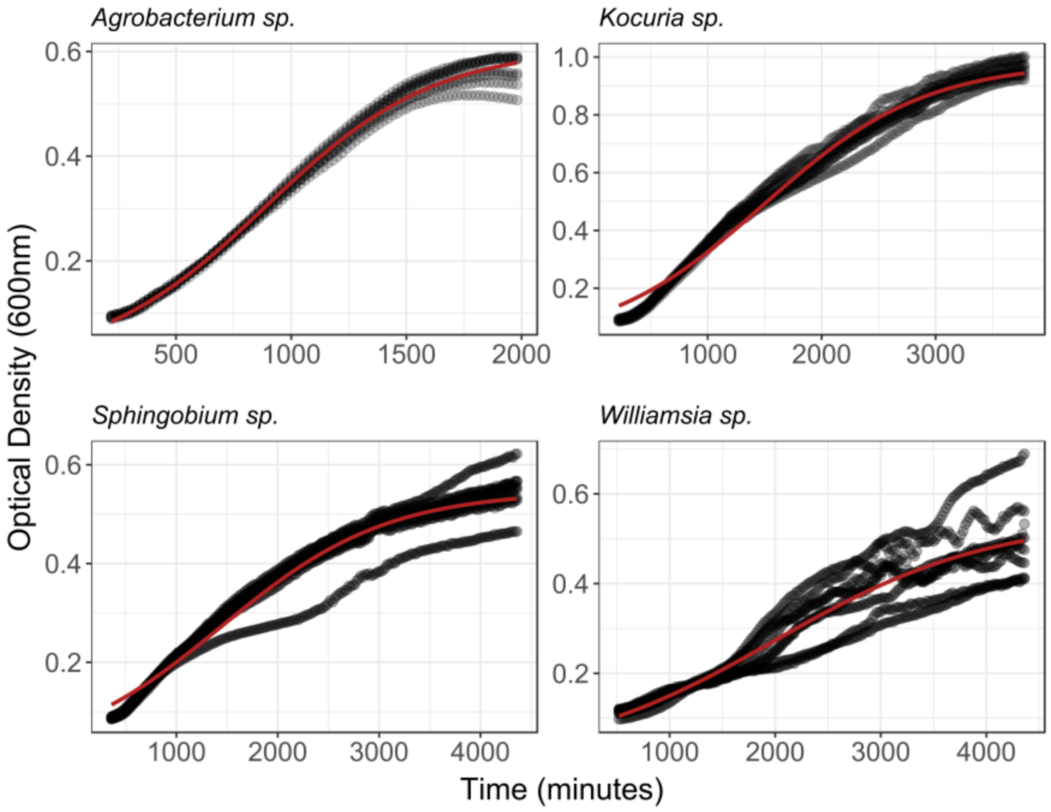

Figure S10. Growth curves of prey species (n=8) and logistic growth fit.

Table S5. Growth rates and carrying capacities of prey species.

**Species**	**r(d^{-1})**	**K (OD)**
Agrobacterium sp.	3.927	0.608
Koccuria sp.	2.035	0.981
Sphingobium sp.	1.765	0.546
Williamsia sp.	1.404	0.544

The predator grows also with approximately 2-4 generations per day, which is comparable with the prey growth rate (Figure S11).

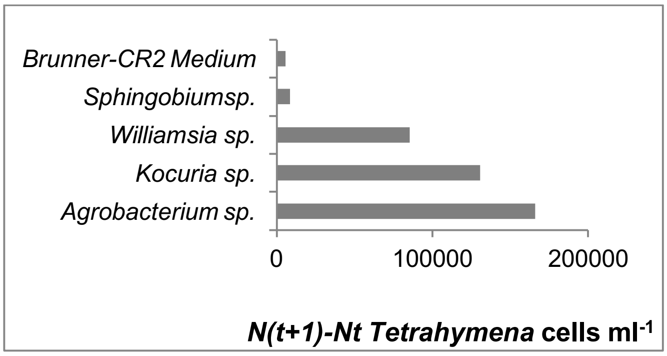

Figure S11. Growth of Tetrahymena sp. with and without prey bacteria.

G. Testing for possible time dependency

In order to evaluate whether our assumption of absence of temporal autocorrelation holds we tested whether relative distances among samples are maintained from one phase to the other. For this, we calculated the pairwise Euclidean distances among all samples at each phase using vegdist() function in the vegan package for R[4]. We then tested whether the pairwise differences differ among phases (i.e. time points) by fitting a linear mixed effect model using lmer() function (lme4[5] R package). Identity of each pair was used as random effect and disturbance phase as fixed effect. To test whether the pairwise differences on average (as estimated from the mixed effect model) changed from one phase to the other we applied t-tests using lsmeans with Bonferroni-Holm correction[6].

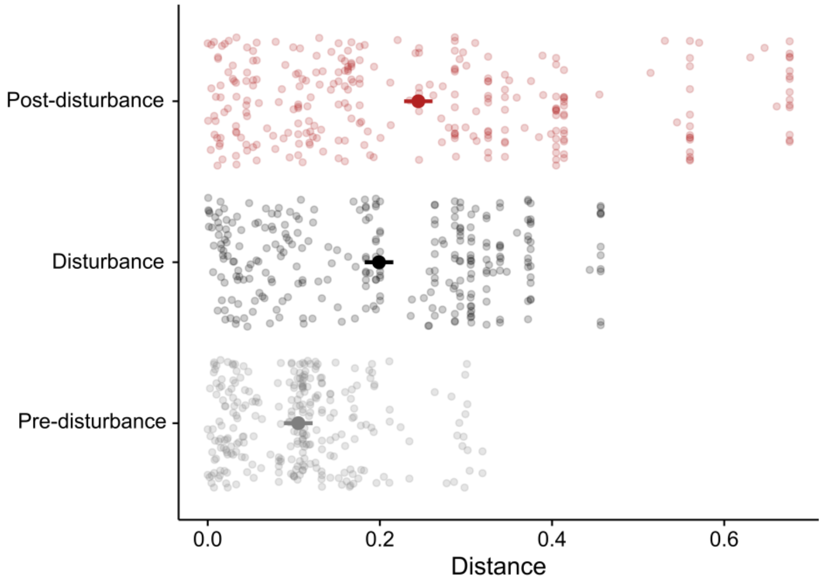

Figure S12: Pairwise Euclidean distances between samples at each phase. Solid points and lines indicate for each phase the estimates from least square means with their confidence intervals.

Table S6: Multiple comparisons of pairwise Euclidean distances between each phase.

Contrast	*df*	*t-ratio*	*p*
Pre-disturbance – Disturbance	552	-9.032	<0.001
Pre-disturbance – Post - disturbance	522	-13.441	<0.001
Disturbance – Post-disturbance	522	-4.408	<0.001

References

1. Saleem, M., Fetzer, I., Dormann, C. F., Harms, H. & Chatzinotas, A. Predator richness increases the effect of prey diversity on prey yield. *Nat. Commun.* **3,** 1305 (2012).
2. Fox, J. W. Testing a simple rule for dominance in resource competition. *Am. Nat.* **159,** 305–319 (2002).
3. Haddad, N. M. *et al.* Species' traits predict the effects of disturbance and productivity on diversity. *Ecol. Lett.* **11,** 348–356 (2008).
4. Oksanen, J. *et al. vegan: Community Ecology Package*. (2016).
5. Bates, D., Mächler, M., Bolker, B. & Walker, S. Fitting Linear Mixed-Effects Models Using lme4. *J. Stat. Softw.* **67,** (2015).
6. Lenth, R. *lsmeans: Least-Squares Means*. (2017).
